# Impact of COVID-19 pandemic on routine immunization

**DOI:** 10.1080/07853890.2021.2009128

**Published:** 2021-12-02

**Authors:** Martin O. C. Ota, Selim Badur, Luis Romano-Mazzotti, Leonard R. Friedland

**Affiliations:** a GSK, Wavre, Belgium; b GSK, Istanbul, Turkey; c GSK, Philadelphia, PA, USA

**Keywords:** COVID-19, pandemic, public health impact, routine immunization, immunization coverage

## Abstract

The current COVID-19 global pandemic continues to impact healthcare services beyond those directly related to the management of SARS-CoV-2 transmission and disease. We reviewed the published literature to assess the pandemic impact on existing global immunization activities and how the impact may be addressed. Widespread global disruption in routine childhood immunization has impacted a majority of regions and countries, especially in the initial pandemic phases. While data indicate subsequent recovery in immunization rates, a substantial number of vulnerable people remain unvaccinated. The downstream impact may be even greater in resource-limited settings and economically poorer populations, and consequently there are growing concerns around the resurgence of vaccine-preventable diseases, particularly measles. Guidance on how to address immunization deficits are available and continue to evolve, emphasizing the importance of maintaining and restoring routine immunization and necessary mass vaccination campaigns during and after pandemics. In this, collaboration between a broad range of stakeholders (governments, industry, healthcare decision-makers and frontline healthcare professionals) and clear communication and engagement with the public can help achieve these goals.

**Key messages**
- The COVID-19 pandemic has a substantial impact on essential immunization activities.
- Disruption to mass vaccination campaigns increase risk of VPD resurgence.
- Catch-up campaigns are necessary to limit existing shortfalls in vaccine uptake.
- Guidance to mitigate these effects continues to evolve.

## Introduction

Widescale infectious disease outbreaks exert considerable direct and indirect effects on a societal, economic and public health level. At present, we remain in the midst of the coronavirus disease 2019 (COVID-19) global pandemic due to severe acute respiratory syndrome-coronavirus-2 [SARS-CoV-2] since its emergence in late December 2019 [1]. As of 1 September 2021, there have been 219 million confirmed cases worldwide and 4.55 million deaths [2]. While the full extent of the health and socio-economic impacts have yet to be measured, clearly the COVID-19 pandemic represents the most challenging public health crisis since the global influenza pandemic of 1918–1919.

In this article, we describe aspects of the current COVID-19, with a particular emphasis on the impact upon global immunization delivery, and highlight the necessary future actions to mitigate the impact of immunization interruption in the most vulnerable populations.

## Impact of pandemics

### Direct impact of the COVID-19 pandemic

Pandemics have direct and indirect effects, although the distinction between these categories can be fluid and often unclear. For the COVID-19 pandemic, direct effects could be considered in terms of impact on health (mortality and immediate and long-term morbidity directly attributable to the SARS-CoV-2 infection) and the direct economic impact; the direct medical costs of treating SARS-CoV-2 and also the broader economic/societal impact of measures taken to reduce/restrict SARS-CoV-2 transmission [3]. The broader economic and societal impacts of such control measures are as yet unknown, and clearly it will be some time before the mortality and morbidity toll of the pandemic is fully understood. While beyond the scope of the present manuscript, some broad observations on mortality/morbidity can be made.

Accurate SARS-CoV-2 mortality estimates are confounded by systemic differences in death registrations and the recognized uncertainty of recording SARS-CoV-2 as a direct cause of death. For example, death in patients with known or clinically suspected SARS-CoV-2 infection may have died due to other factors, including underlying comorbidities, exacerbated by SARS-CoV-2. Using “excess deaths” as a surrogate estimate provides an alternative approach, where the overall number of deaths or all-cause mortality rates in a particular period during the pandemic are compared to similar historical periods [4,5]. SARS-CoV-2 mortality rates vary widely in different countries and are influenced by population demographics, principally the age structure [6–8]. SARS-CoV-2 mortality is greatest in vulnerable/at risk populations; chiefly the elderly (≥65 years), and patients with medical comorbidity [9–11]. However, this is far from absolute e.g. United States (US) data reports substantial deaths due to SARS-CoV-2, and also substantial excess mortality in younger adults aged 25–44 years [12]. While fatality in children is low [13], one broader concern would seem to be the development of a multisystem inflammatory syndrome in children (MIS-C), similar to Kawasaki disease, associated with paediatric SARS-CoV-2 infection [14,15]. Ethnicity and occupation also influence infection rates and mortality. Data from countries with substantial multi-ethnic populations indicate a greater risk of infection, hospitalization and death in non-Caucasian ethnic groups (i.e. African, Hispanic and Asian origin) [16–18].

Healthcare workers (HCWs) are also disproportionately at risk of infection, a feature seen in recent previous pandemics with 18–21% of all Middle East respiratory syndrome (MERS) and severe acute respiratory syndrome (SARS) cases occurring in HCWs [19], while HCWs accounted for substantial number of cases and mortality in the Ebola outbreaks in West Africa [20,21]. SARS-CoV-2 infection and mortality rates in HCWs are disproportionately higher [22–24], and throughout the present COVID-19 pandemic, shortage of HCWs due directly to SARS-CoV-2 infection or as a self-isolation precaution as a result of infection in close-contacts has further impacted healthcare delivery.

### Indirect impact of the COVID-19 pandemic

Indirect effects can be broadly considered as the negative impact of the social measures implemented to reduce SARS-CoV-2 infection, along with the necessary prioritization of healthcare services directed towards SARS-CoV-2 care; together these have inevitably led to substantial disruption of routine medical services.

While measures have been widely implemented in most counties, the scope and scale and their nature and timing vary substantially; reflecting national or more local transmission and disease activity patterns. Most countries have initiated a national lockdown, of some form and at some time, restricting people’s movement except those providing essential services (“key workers”). The general public had far more limited activities, and often only movement for essential actions such as food/grocery shopping and for non-routine medical care. Educational activities have been subject to substantial reorganization of teaching, and there has been widespread and far-ranging effects upon commercial activities, with substantial economic hardship to individuals, companies and employees. These measures have had some effect on reducing any escalation of transmission and mortality [25–27], although when eased or lifted, there has often been a notable increase in SARS-CoV-2 cases and mortality [28,29].

Such restrictions have many downstream effects. For example, even when medical services are available, people may be unable to access them due to transport interruptions, economic hardship and reluctance to leave home or attend medical facilities out of caution or fear of SARS-CoV-2 exposure. HCWs may experience similar challenges and concerns, a feature evident in the early pandemic phase when access to protective equipment was unreliable and erratic.

The full extent of disruption on routine services and its impact on health and early deaths is as of yet unknown. From a global perspective, an extensive World Health Organization (WHO) survey during the initial pandemic phase reported substantial reductions in all routine health services, with a 70% reduction in routine immunization services, considerable reduction in access to essential malaria, tuberculosis (TB) and HIV services [30]. Patient reluctance to travel to their health service providers out of fear of SARS-CoV-2 transmission is a further consideration [31]. Modelling studies estimate that disruption to essential malaria, TB and HIV services in countries with high disease burdens would lead to notably greater mortality over the next 5 years (malaria by 36%, TB by 20%, and HIV by 10%) [32]. Recent data from South Africa shows that although existing HIV treatment provision was maintained, both HIV testing and initiation of retroviral therapy fell substantially during the initial lockdown (each by over 40%), with some recovery following easing of restrictions [33]. Other modelling studies forecast that reduction in essential childhood healthcare in LMICs would lead to, at a conservative estimate, an increase in mortality in children aged <5 years of 9.8% each month, with much of the projected impact directly attributable to deaths due to vaccine-preventable disease (VPD) resulting from immunization service disruption [34].

It should be recognized that declines in VPDs transmitted *via* the respiratory route during the current SARS-CoV-2 pandemic have been observed. Data from the Invasive Respiratory Infection Surveillance (IRIS) network indicates substantial declines in invasive meningococcal disease, invasive pneumococcal disease (IPD) and *Haemophilus influenzae* type b disease in 2020 compared to 2018–2019 [35], and other surveillance data from the United Kingdom (UK), the Netherlands and South Korea all indicate reduced IPD notifications in the April-June 2020 period compared to previous years [36–38], while most surveillance data indicate that influenza activity is lower since the COVID-19 pandemic onset [39–42]. These findings would suggest that social restrictions to mitigate SARS-CoV-2 transmission have had, at least in the short-term, a beneficial impact on some important VPDs (although we can also speculate that this reduction may also reduce natural immunological boosting within the population, and so lower herd immunity). Nevertheless, grave concerns exist regarding the broader long-term impact of immunization disruption during the COVID-19 pandemic, and the subsequent threat of VPD resurgence.

## Disruption to routine immunization services

Throughout the pandemic the WHO issued regular news bulletins on the current status of global immunization activities and regular and evolving guidance on routine and mass immunization policies. Early guidance to halt mass vaccination campaigns towards measles, polio and diphtheria, an essential component of activities in lower income countries, raised substantial concerns, putting an estimated 80 million infants at risk of these diseases [43], although subsequent guidance from WHO and other agencies to restart such activities have led to an albeit non-linear continuation of immunization campaigns at a country (or more local) level when pandemic circumstances allow [44,45].

Guidance for routine immunization activities was issued as early as March 2020 [46–48]. These all recommended (and continue to do so) that routine immunization activities for all vaccine-eligible individuals remain a priority and should be maintained wherever possible, within the broader context dictated by local circumstances and COVID-19 responses. Childhood vaccination remains paramount; newborns should receive recommended vaccines (e.g. against hepatitis B) within maternity units; infants and younger children should receive all scheduled vaccinations relevant to their age milestones, with priority given for completion of all primary series vaccines (in particular use of combination vaccines covering measles, rubella and polio). Guidance emphasized the importance of catch-up activities when scheduled vaccinations are delayed. In addition, when feasible, maternal immunization and pneumococcal and seasonal influenza vaccination for vulnerable individuals should continue as planned [46–48]. While such guidance is clear, widespread service disruption has been a feature throughout much of the pandemic.

### Reported delays and shortfalls in routine immunization services

Available data indicate that routine immunization services were affected in most countries. An early indicator of impact is changes in routine vaccine ordering by national or regional authorities, whereby compared with 2019 patterns, vaccine orders declined early in the pandemic, with considerably lower orders in the mid-March to mid-April 2020 period seen in the US [49] and in Europe [50,51].

Substantial declines in a range of scheduled immunizations were reported across numerous countries early in the pandemic (Table 1). In the US, a nationwide reduction in measles vaccination was apparent, with approximately 80% fewer doses given to those aged >24 months in mid-March to mid-April 2020 compared to January and February 2020 data (and 50% fewer doses in those aged ≤24 months) [49]. Regional data analysis (from Michigan) found a 15% reduction in non-influenza vaccine doses in those ≤24 months during January–April 2020 compared to the same periods in 2018–2019, and also that less than 50% of infants were up-to-date with all recommended milestone vaccines in May 2020 [52], while in Alabama immunization rates in all individuals ≤18 years fell by over 50% [53]; a nationwide survey from the US Vaccines for Children Program also reported substantial declines in paediatric immunization rates in March and April 2020 compared to corresponding 2019 rates across all US states [54]. Specific concerns remain regarding administration of measles containing vaccines (MCV1 and MCV2) in children in the US [55]. Data from California also report substantially fewer infant immunization visits and substantial disruptions in infant and older child immunization rates, with a particular impact on MCV administrations [56], while childhood MCV dosing declined by 54.7% in Alabama [53].

**Table 1.** Initial disruption to routine immunization uptake in selected countries.

Region	Country	Period	Reduction in vaccine uptake^a^
North America			
	United States	Mid-March–mid-April 2020	MCV1 (50–80%) [49]
Europe			
	United Kingdom	Mid-March–mid-April 2020	MMR (19.8%) [57]
	Spain	Mid-March–mid-April 2020	All routine infant vaccinations (8–20% relative decline) [59]
Latin America			
	Brazil	March–April 2020	MMR (27%); DTP-HBV-/Hib (18%); polio (18%) [79]
Asia			
	Pakistan	Mid-March–mid-May 2020	All routine childhood vaccinations (50% relative decline) [68]
	India	April 2020	Number of fully immunized infants (87% relative decline) [75]
	Singapore	January–April 2020	MMR (>25%); DTPa-HBV-IPV/Hib (10%); PCV (>10%) [65]
Africa			
	Angola/Senegal	April–June 2020	DTP3 (>10%) [80]
	Burundi/Gabon/ Guinea/ Nigeria	April–June 2020	MCV1 (>10%); DTP3 (>10%) [80]

^a^Reduction compared with previous historic trends.

DTPa-HBV-IPV/Hib: combined diphtheria-tetanus-acellular pertussis-hepatitis B-inactivated poliovirus-Haemophilus influenzae type b conjugate vaccine; DTP-HBV-/Hib: combined diphtheria-tetanus-acellular pertussis-hepatitis B-Haemophilus influenzae type b conjugate vaccine; DTP3: diphtheria–tetanus–pertussis vaccine (dose 3); MCV1: measles-containing vaccine (dose 1); MMR: measles–mumps–rubella vaccine; PCV: pneumococcal conjugate vaccine.

Similar patterns were reported across Europe. In the UK, routine MMR vaccinations in infants aged <1 year in England during the first 3 weeks of the national lockdown (implemented on 23 March 2020) were 19.8% lower than for the same period in 2019, although vaccination counts subsequently increased, and were broadly comparable to 2019 numbers by the end of April 2020 [57]; similar trends were seen in Scotland [58]. In Spain, reductions in the number of routine infant vaccinations ranged from 8% to 20% [59], and German data indicates that while scheduled child and adult vaccination visits fell in early lockdown, most were later fulfilled, although up to 20% of children and 40% of adults were without re-scheduled catch-up appointments [60]. In Italy, early reports indicated that the number of vaccine doses administered in Rome fell by 16% over the first 10 weeks of lockdown [61], and subsequent regional and national surveys report reductions in both mandatory and non-mandatory childhood vaccinations [62,63]. In the Netherlands, the number of infants receiving the first MMR vaccination dose (given at 14 months) initially dropped by 6–14%, although this shortfall was subsequently addressed by a catch-up campaign [37].

In Asia, while some countries such as South Korea reported little effect on paediatric immunization [64], others reported interrupted services. In Singapore, over the January–April 2020 period, childhood MMR uptake rates in selected primary care, hospital and private paediatrician clinics fell by 25.6–73.6%, a pattern which if projected to the overall population, would translate as 84% MMR vaccine coverage in children aged ≤2 years, below the 95% target required for herd immunity [65]. Oher vaccine uptake also fell; hexavalent vaccine (by up to 10.3%) and pneumococcal conjugate vaccine uptake (between 8.0–67.8%) compared to 2019 data [65]. In Japan, survey data also indicates a decline in childhood measles and rubella vaccine uptake during lockdown [66]. A recent large-scale immunization impact appraisal surveying 19 countries in the Southeast Asia and Western Pacific regions reported disruptions in countries, impacting most immunization targets in the period up to June 2020 (although Australia and South Korea were less affected than other surveyed countries). A key finding was that substantial socio-economic, ethnic and geographical disparities exist, with the impact greatest in rural and poorer communities [67].

Large-scale studies monitoring activities in Pakistan and in India report substantial declines. In Pakistan, data for the Sindh province (comprising approximately 48 million people) shows an initial 50% relative decline in all routine childhood scheduled vaccinations during lockdown (from 23 March–9 May 2020), although rates subsequently increased during late May–June 2020 and to some extent compensated for the earlier shortfall [68]. A similar pattern was seen for Karachi, Sindh’s major city, where following an initial decline, 62% of those children who had missed scheduled vaccinations were immunized *via* catch-up activities by September 2020 [69,70]. Although oral polio vaccination (OPV) activities were initially suspended, mass vaccination programs were successfully implemented between August–October 2020, despite substantial challenges (with over 32 million children vaccinated in late October 2020) [71,72]. While this may address concerns about re-emergence of polio in the region, in neighbouring Afghanistan only 2 out of 10 planned polio vaccination campaigns have taken place, both prior to the pandemic [73]. This is alarming and may have contributed to the recently reported increase in “Wild virus type 1” confirmed polio cases [74]. In India, data from Rajasthan province (where lockdown restrictions were greatest between March and May 2020) indicates an 87% relative decline in the number of fully immunized infants aged 9–11 months in April 2020 compared with pre-lockdown levels, although this was then somewhat compensated by the relative 23% increase in May 2020 [75]. However, while nationwide data shows a similar decline (69% in April 2020), there was no discernible recovery, with rates remaining lower in May and June 2020 (by 23% and 31% respectively) [75]. It remains to be seen how the more recent devastating upsurge in the COVID-19 pandemic in India has impacted on current immunization patterns.

Similar declines are seen elsewhere. For example, in Lebanon, a 47% decline in public sector immunization delivery was reported in Lebanon for March 2020 compared to 2019 levels (including a 73% decline in measles vaccination and 48% decline in OPV immunization) [76], while in Saudi Arabia routine recommended infant vaccinations in Riyadh declined by over 70% in March–May 2020 compared to recent historical administration rates, with delays in receipt of recommended childhood vaccinations reported in other regions [77,78]. In Brazil, national data shows a reduction in key infant vaccination dosing in March/April 2020 (when restrictions were greatest) compared to previous years; dose 3 for pentavalent and polio vaccines given at 6 months of age declined by 18% and MMR dosing at 12 months was 27% lower. While rates subsequently improved in May/June, these have not accounted for the earlier shortfall [79]. Ancillary Brazilian data shows that while 20% of children ≥2 months had missed scheduled vaccinations, disparities exist; missed immunization was more common in poorer families, and more common in the Northern Amazon region and less common in the Southern regions [79].

For Africa, ascertaining pandemic impact on immunization rates is challenging, in part, as electronic immunization registries are often lacking. Perhaps the most complete data are that reporting on routine childhood immunization coverage across 15 countries for the April–June 2020 period; assessing uptake of the diphtheria, tetanus and pertussis (DTP) vaccine first and third dose (DTP1 and DTP3) and also MCV1 and MCV2 doses compared to 2018–2019 data [80]. This study included countries with high pre-pandemic coverage and others with relatively lower coverage; six (Angola, Burundi, Gabon, Guinea, Nigeria, and Senegal) had >10% decline in DTP3 uptake, while four (Burundi, Gabon, Guinea, and Nigeria) reported >10% decline in MCV1 uptake. A general observation was that those countries with previously high immunization rates had relatively little decline in uptake compared to 2019 rates, while those with historically lower rates had larger declines, although this was not absolute and varied depending upon vaccine [80]. Data for North Africa is more limited; a survey of immunizing paediatricians in Morocco found that most scheduled childhood vaccines were delayed during the initial pandemic phase, chiefly due to parental concerns in attending clinics [81].

## Consequences of disrupted immunization services

Clearly immunization disruption occurred during the initial phase of the COVID-19 pandemic in most countries. While some element of catch-up was evident, we await more complete and extended data reporting to fully understand the overall impact; as the pandemic continues, it seems likely that disruption may continue, and it may be some time before the complete picture emerges.

These general observations raise concerns that much of the public health gains realized by immunization will be lost. Estimates indicate that each year immunization programs prevent 2.7 million cases of measles, 2 million cases of neonatal tetanus, 1 million pertussis cases, 600,000 cases of poliomyelitis, and 300,000 diphtheria cases [82]. Much of this benefit is achieved through high infant immunization rates (*via* routine immunization along with supplementary immunization activities [SIAs] implemented as local or more widespread national mass vaccination campaigns), providing direct protection and a broader indirect protection through herd immunity. Immunization service disruption threatens to reverse hard-won progress on VPDs (especially if present immunization coverage falls below that required to sustain and support herd immunity. Necessary coverage is influenced by organism specific transmission factors, notably the basic reproductive rate (*R*_0_); the higher the *R*_0_, the higher the vaccine coverage necessary for infection control at the population level) [83]. This is particularly important for infections such as measles and pertussis (high *R*_0_ of 12–18 and 12–17 respectively) and where necessary vaccine coverage for herd immunity is higher (e.g. 92–94% for measles) than infections with lower transmission rates such as diphtheria (*R*_0_ of 6–7) [83,84]. In contrast, although precise estimates remain incomplete, the SARS-CoV-2 *R*_0_ is generally considered lower, with correspondingly lower herd immunity thresholds required to stop community transmission [85].

While addressing the COVID-19 pandemic is an immediate concern, we must remain vigilant of other pathogens. An early modelling study (from a pan-Africa perspective) estimated that continuing routine immunization would avert a far greater number of lives lost due to VPDs compared to every single potential death due to SARS-CoV-2 averted with suspension of routine immunization services [86]. Concerns about VPD resurgence – in particular measles – but also diphtheria and polio have been raised and widely reported in some detail [87–89]. These reflect pre-existing apprehension that pre-pandemic immunization rates had fallen below the necessary herd immunity threshold in many countries, with numerous outbreaks in many regions observed prior to the pandemic. One sobering statistic is that, prior to COVID-19, measles mortality was at a recent historical high, with over 200,000 deaths in 2019 [90]. Halting planned mass vaccination campaigns such as planned SIAs targeting measles in Kenya [91], along with interruption of routine programs due to COVID-19, only exacerbates such concerns. Maintenance of existing vaccine coverage rates against such pathogens, and where necessary, restoration to pre-pandemic levels when rates have declined is essential, albeit challenging while the pandemic continues.

## Guidance for immunization during and after pandemics

**Figure 1.**
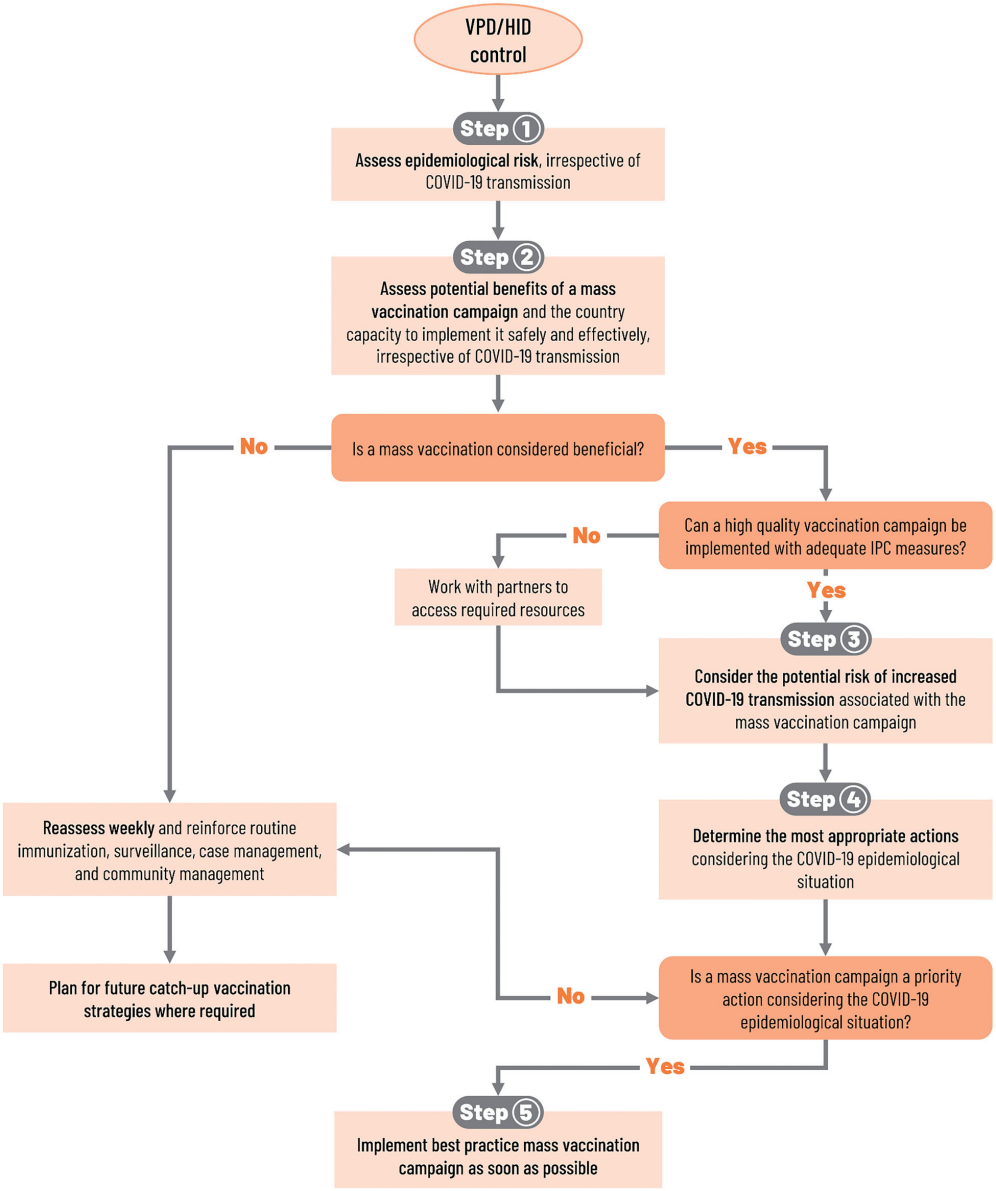
Decision-making framework for implementation of mass vaccination campaigns: Decisions should follow a step-by-step sequence. Modified from WHO published guidance [44]. COVID-19: coronavirus disease 2019; HID: high impact disease; IPC: infection prevention and control; VPD: vaccine-preventable disease.

Guidance on immunization activities have evolved throughout the COVID-19 pandemic, and continues to do so. While initial guidance was to halt planned mass vaccination campaigns (which had some impact) [43], current guidance is to proceed with such campaigns, when and where local circumstances allow [44]. A graphic overview of the current guidance is presented in Figure 1. Clearly an important consideration is the potential for greater COVID-19 transmission during mass vaccination activities. The risk–benefit ratio is complex, and in light of the continued pandemic, it will be necessary and prudent to tailor activities within the context of local circumstances.

Similarly, routine immunization should continue [46,47], integrated with catch-up activities to ensure vaccination at the earliest opportunity of those eligible individuals who missed immunization due to service disruptions [92]. The WHO emphasizes the importance of maintaining existing routine immunization budgets [93], providing guidance on delivering routine services in the context of the ongoing pandemic [94]. Global guidance is complemented by guidance issued by stakeholders, national advisory bodies and societies [48,95–98], all of which describe the core principles governing the necessity for maintaining routine immunization, with recommendations and proposals on how best to do so. Again, these emphasize the need to identify all who have missed scheduled vaccinations and implementing necessary catch-up activities. Such activities should also address any disparities in access experienced by racial/ethnic groups or where driven by economic circumstances [99,100]. What remains somewhat unclear is how the roll out of SARS-CoV-2 vaccination will impact on this, and how SARS-CoV-2 immunization can be integrated alongside routinely recommended adult vaccines. At present, there is limited information on potential co-administration effects; usually SARS-CoV-2 vaccines should be given at least 14 days prior to or after any other vaccines (although this is not absolute); for example, the CDC now allows coadministration of SARS-CoV-2 vaccines with other routinely recommended vaccines [101].

## A call to action for future pandemics

Since the COVID-19 pandemic was declared in March 2020, we have learned many important lessons that should inform future pandemic preparedness plans to help mitigate the many risks and challenges we have faced, including minimizing the impact on routine immunization programs. Vaccine hesitancy should continue to be addressed, as its impact is not limited to pandemic vaccines but continues to extend to routinely recommended vaccines [102,103]. We continue to learn how to leverage the increased awareness around the COVID-19 vaccination campaigns and expand these call-to-action messages to routine immunizations, and also how potential use of novel strategies to increase COVID-19 vaccine uptake (such as offering incentives) could also be applied for routinely recommended vaccines [104,105]. Clear communication between public health authorities with providers and the general public, and from providers to parents/caregivers on the importance, value and safety of routine immunization will remain a critical piece to help alleviate concerns and address vaccine hesitancy. Besides providers, it will be important to identify and engage with leaders in the community that may help resonate public health messages related to the value of routine vaccines, especially when the discussion around public health becomes tainted with political and/or non-medical aspects. The influence of social media, digital platforms and access to information/misinformation is likely to continue to expand, and we must continue to study the complex behavioural mechanisms behind these evolving networks and digital communities and learn how social media may be best used to improve health literacy and foster public trust in vaccination [106,107].

Finally, we do not need to wait for the start of a future pandemic to continue our efforts and exploring new approaches on raising awareness of the public health benefits of vaccination, and while most of the disruptions we describe relate to childhood immunization, a broader perspective also applies. As the world population gets older, the overall positive impact of adult vaccination on health and wellbeing raises important questions and opportunities to make sure this twenty first century “older” adult population will also translate into an “older and healthy” population [98]. An online survey conducted during July and August 2021 among 16,000 adults aged ≥50 years across eight countries (USA, Italy, Spain, Germany, France, Brazil, Canada, and Japan) looking at attitudes to health and ageing showed improved attitudes to vaccination due to COVID-19; while 44% of respondents indicated the importance of staying up to date with recommended vaccines prior to the pandemic, this proportion then increased to 65% of respondents after the pandemic onset [108]. Good health was significantly more important to this age group for their quality of life over the next 10 years than financial security. While eight in ten of those surveyed reporting being vaccinated for COVID-19, only 19.8% believed they were up to date on all recommended adult vaccines. According to those surveyed, vaccination rates can be improved with clearer, more consistent information on recommended vaccines (including the rationale for use and safety aspects) allied with easier access to vaccination services [108]. These aspects should underpin future immunization efforts.

## Conclusions

The current COVID-19 pandemic has exerted a substantial toll beyond the direct effects of SARS-CoV-2 morbidity and mortality; chiefly social and economic disruption and in its impact on routine healthcare services including routine immunization activities. Immunisation rates have fallen substantially during the pandemic, placing individuals and communities at greater risk of VPD. Addressing existing shortfalls and strengthening routine services is essential to avoid routine immunisation becoming another casualty of the pandemic. Collaboration between a broad range of stakeholders (governments, industry, policy-makers and frontline HCWs) allied with clear public communication and engagement to restore confidence towards increasing routine vaccine uptake and necessary catch-up activities can help achieve these goals. A plain language summary of our observations is presented in Supplementary Figure 1.

## Supplementary Material

Supplemental Material
