# Supplementary figures and images for: Impact of COVID-19 pandemic on routine immunization
Source: Ann Med. 2021 Dec 2;53(1):2286–97. doi: 10.1080/07853890.2021.2009128 (PMC8648038; doi:10.1080/07853890.2021.2009128)

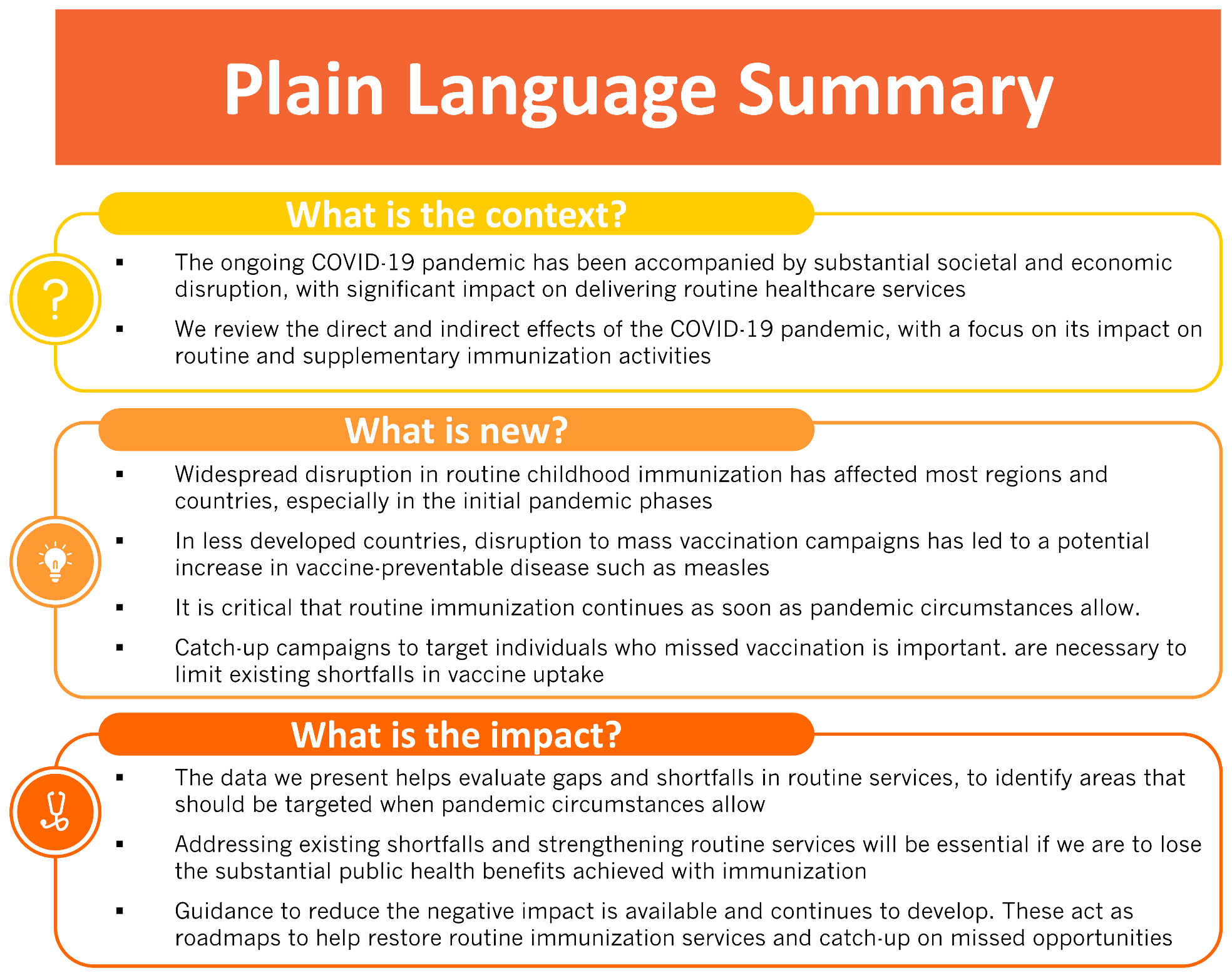

Supplement: Supplemental Material [file IANN_A_2009128_SM4867.tif]
